# A Race Against Time—Changing the Natural History of CRIM Negative Infantile Pompe Disease

**DOI:** 10.3389/fimmu.2020.01929

**Published:** 2020-09-04

**Authors:** Punita Gupta, Brian J. Shayota, Ankit K. Desai, Fuad Kiblawi, Dorothy Myridakis, John Messina, Peter Tah, Lorien Tambini-King, Priya S. Kishnani

**Affiliations:** ^1^St. Joseph's University Hospital, Paterson, NJ, United States; ^2^Texas Children's Hospital, Balor College of Medicine, Houston, TX, United States; ^3^Duke University Medical Center, Durham, NC, United States

**Keywords:** infantile Pompe disease, CRIM negative, early diagnosis, immunomodulation, newborn screening, anti-drug antibodies

## Abstract

We report the clinical course of the first prenatally diagnosed cross-reactive immunologic material (CRIM)-negative infantile Pompe disease (IPD) patient [homozygous for c.2560C>T (p.Arg854X) variant in the *GAA* gene] to undergo prophylactic immune tolerance induction (ITI) and enzyme replacement therapy (ERT) within the first 2 days of life. Both parents were found to be carriers of the c.2560C>T (p.Arg854X) variant through prenatal carrier screening. Fetal echocardiogram at 31 weeks of gestation showed left ventricular hypertrophy. An echocardiogram on the 1st day of life revealed marked biventricular hypertrophy. Physical exam was significant for macroglossia and hypotonia. A short course of Prophylactic ITI with rituximab, methotrexate, and intravenous immunoglobulin (IVIG) in conjunction with ERT at a dose of 20 mg/kg every other week was started on day 2 of life. The patient completed the ITI protocol safely and complete B-cell recovery, based on CD19 count, was noted by 3 months of age. The patient never developed anti-rhGAA IgG antibodies to ERT. Vaccinations were initiated at 9 months of age, with adequate response noted. Complete recovery of cardiac function and left ventricular mass was seen by 11 weeks of age. At 8 months of age, the patient developmentally measured at 75–90% on the Alberta Infant Motor Scale, walked at 11 months and continues to develop age-appropriately at 50 months of age based on the Early Learning Accomplishment Profile. ERT dosing was increased to 40 mg/kg every 2 weeks at 32 months of age and frequency increased to 40 mg/kg every week at 47 months of age. Patient continues to have undetectable antibody titers, most recently at age 50 months and urine Hex4 has remained normal. To our knowledge, this is the first report of successful early ERT and ITI in a prenatally diagnosed CRIM-negative IPD patient and the youngest IPD patient to receive ITI safely. With the addition of Pompe disease to the Recommended Uniform Screening Panel(RUSP) and its addition to multiple state newborn screening programs, our case highlights the benefits of early diagnosis and timely initiation of treatment in babies with Pompe disease, who represent the most severe end of the disease spectrum.

## Introduction

Pompe Disease is an autosomal recessive glycogen storage disorder caused by a deficiency of the lysosomal enzyme acid alpha-glucosidase (GAA), resulting in progressive glycogen accumulation. Patients with a severe deficiency of GAA activity present in infancy with cardiomyopathy and skeletal myopathy. The diagnosis of infantile Pompe disease (IPD) is often delayed, the median age of diagnosis is 4.7 months since non-specific findings like cardiomegaly, respiratory distress, hypotonia, and failure to thrive do not typically present until 2 months of age, although signs of the disease are present at birth (1). Without enzyme replacement therapy (ERT) with recombinant human acid alpha-glucosidase (rhGAA), death is imminent, usually within the first 2 years of life secondary to cardiorespiratory failure (2). Treatment with ERT has resulted in significantly improved survival, yet long-term consequences of the disease like facial muscle weakness, speech disorders, and dysphagia as well as signal alterations of the deep white matter on brain MRI are now being recognized (3, 4).

Despite the improved clinical outcomes, the response to ERT is very heterogeneous. Various factors known to impact the response to ERT include age on ERT initiation, extent of preexisting pathology, degree of muscle damage, cross-reactive immunologic material (CRIM) status, and anti-rhGAA IgG antibodies (5). While ERT has changed the natural history of Pompe disease and has significantly improved the overall survival of patients with IPD, it is not able to reverse the underlying pathology. Prior studies in patients diagnosed via newborn screening and treated with ERT have demonstrated that even a delay of few days in treatment initiation can impact the long-term outcomes of patients with IPD (6). It is of utmost importance to initiate treatment prior to irreversible muscle damage in such a rapidly progressive disease.

CRIM status is determined based on a patient's endogenous GAA enzyme level which is influenced by the nature of pathogenic variants. In CRIM-negative patients, there exist two deleterious *GAA* mutations, which lead to absence of native GAA enzyme production and, therefore, lack of exposure of the developing immune system to the GAA protein. Consequently, these patients are not immune tolerant to GAA and mount a high and sustained antibody response to rhGAA. Often these responses neutralize either enzyme uptake into cells or the catalytic activity of the enzyme (5). Thus, it is not surprising that high and sustained antibody titers (HSAT) herald clinical decline in CRIM-negative patients, who are at the most severe end of the disease spectrum (7, 8). Despite advances in ERT treatment, cases of CRIM-negative IPD treated with ERT alone still result in invasive ventilation or death within the first 3 years of life (9, 10).

The management of CRIM-negative IPD has evolved significantly in recent years with the advent of immune tolerance induction (ITI), which helps reduce the immune response to ERT, but to date has not been administered early in the neonatal period. Initiation of ERT soon after birth in IPD is the goal as a delay of just 10 days has been associated with worse biological, physical, and developmental outcomes (6, 11). We present the first case of a prenatally diagnosed CRIM-negative IPD patient to undergo prophylactic ITI and ERT with recombinant human GAA within the first 2 days of life, who at 50 months of age is meeting all her developmental milestones and attending a regular prekindergarten class.

## Methods

*GAA* mutation analysis on the amniocentesis sample was done at Bioreference Laboratories (Elmwood Park, NJ). Anti-rhGAA IgG antibody titer measurements were performed at Sanofi Genzyme Corporation (Cambridge, MA). Postnatal *GAA* variant analysis and urinary glucose tetrasaccharide biomarker (Glc_4_/Hex_4_) measurements were performed at the Duke University Hospital Biochemical Genetics Laboratory. T and B cell studies and immunoglobin titers were performed at Mayo Clinic Laboratories (Rochester, MN). The patient's mother provided informed consent for the use of clinical data and images for publication. Data for CK levels, anti-rhGAA IgG antibody titers, AST and ALT from birth to 170 weeks were extracted and analyzed.

**Figure 1:**
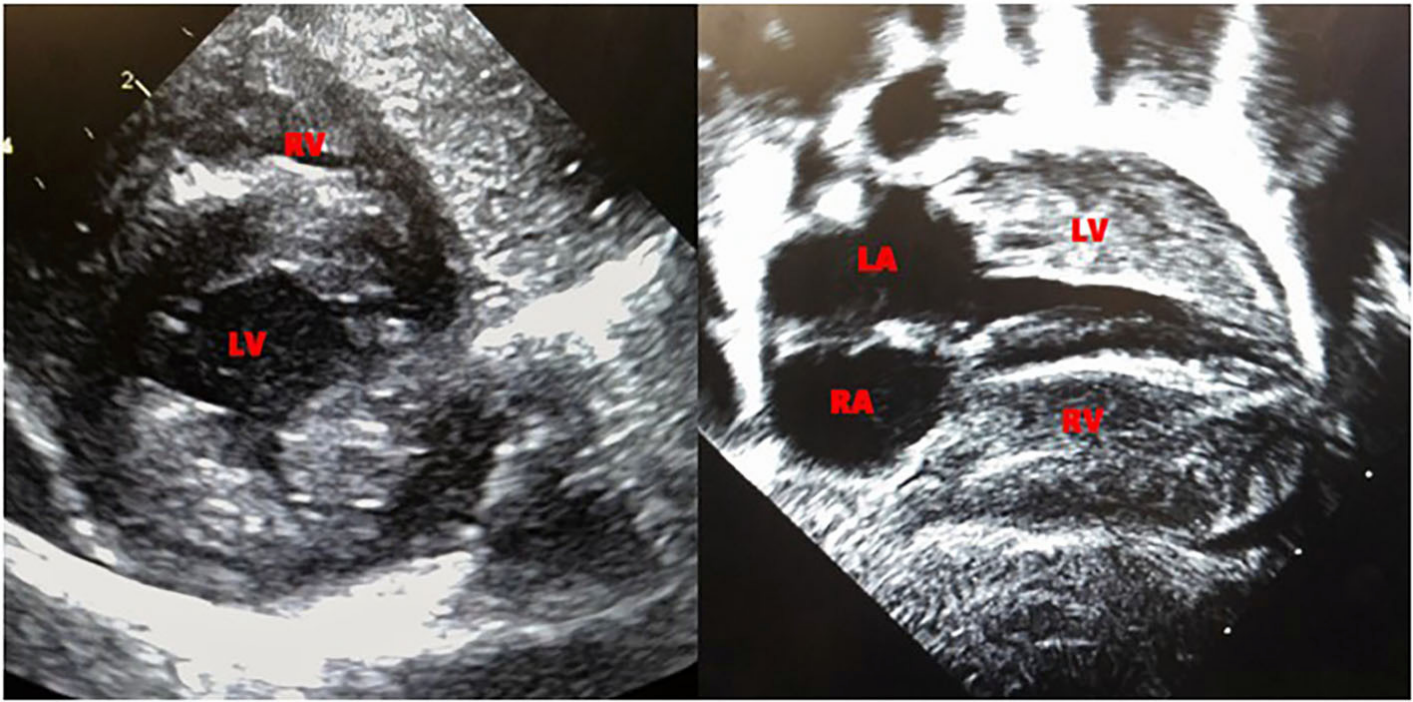
Echocardiogram at 6 days of life, showing significant biventricular hypertrophy.

The ITI approach that included four doses of weekly rituximab (375 mg/m^2^, intravenously), three cycles of methotrexate (0.4 mg/kg; three doses per cycle with first three ERT infusions, subcutaneously or orally), and monthly IVIG (500 mg/kg) was initiated along with ERT, as described previously (Supplemetary Figure 1) (12, 13). The limited dataset on our patient has been previously published as part of a large cohort (13).

## Case Presentation

### Diagnosis

The patient is a 4-year-old CRIM-negative IPD patient diagnosed prenatally. Family history was significant for the demise of their first child at 2 days of life in the Dominican Republic, from cardiorespiratory failure of unknown etiology. Both parents are of Dominican Republican ancestry and are second cousins once removed. The mother of the patient underwent prenatal carrier screening, which revealed that she was a carrier of the c.2560C>T (p. Arg854X) variant in the *GAA* gene. The father was subsequently found to be a carrier of the same variant. Amniocentesis performed at 19 weeks gestation revealed a fetus with a 46, XX karyotype, and homozygous (c.2560C>T) *GAA* gene variant, confirming CRIM-negative IPD (14). Fetal ultrasound at 28 weeks gestation was negative for any obvious birth defects or cardiomyopathy. However, a fetal echocardiogram performed at 31 weeks gestation showed mild left ventricular hypertrophy affecting the interventricular septum.

### Clinical Course in the Neonatal Period

The patient was born via repeat cesarean section to a 31-year-old G3P2001 mother at 38 weeks gestation. APGARS were 9 and 9 at 1 and 5 minutes, respectively and birth weight was 3.5 kg. The physical exam was significant for macroglossia and hypotonia. There was no hepatosplenomegaly. Postnatal molecular studies confirmed the homozygous c.2560C>T variant.

#### Respiratory Status

The patient was initially on room air, but after a few hours she had some desaturations and tachypnea to 80 breaths/min and was started on CPAP. Chest X-ray on the 1st day of life showed-submaximal expansion of lungs, the possibility of cardiomegaly could not be ruled out. CPAP was required intermittently, finally discontinued by week three of life, and she has remained on room air since.

#### Gastrointestinal Status

The patient was initially kept NPO and on parenteral nutrition due to tachypnea. Trophic feeds were started via oral gastric tube (OGT) on the 4th day of life but she developed abdominal distension and was made NPO again. OGT feeds were restarted on the 7th day of life. On day eight of life, she was noted to have abdominal distension, greenish aspirates, and irritability. Abdominal X-ray revealed dilated bowel loops. Upper gastrointestinal endoscopy was normal. Feeding evaluation was requested and she was found to have normal suck-swallow coordination. Gradually oral feeds were restarted and she was taking full feeds by mouth by the 19th day of life.

#### Cardiac Status

Echocardiogram performed on day 6 of life revealed biventricular hypertrophy with prominent moderator band and muscle bundles (Figure 1). The left ventricular mass index (LVMI) was 65.4 gm/m^2^ (15). Electrocardiogram showed normal sinus rhythm and biventricular hypertrophy. On 12th day of life, the patient was noted to have mild periorbital and pedal edema, the brain natriuretic peptide (BNP) rose from 556 to 778 pg/ml (normal range 1–100pg/ml) and echocardiogram showed a mild decrease in ejection fraction from the previous study with LVMI of 66 gm/m^2^. Ionotropic treatment with milrinone was initiated. The echocardiogram on the 14th day of life revealed a LVMI of 69 gm/m^2^. The periorbital and pedal swelling resolved, cardiac function improved over the next few days and BNP came down to 129 pg/ml. Echocardiogram on the 22nd day of life showed a LVMI of 65 gm/m^2^.

### Treatment Details

Prophylactic immune tolerance induction (ITI) was started on day 2 of life with a short course of IV rituximab, SC methotrexate, and intravenous immunoglobulin (IVIG) (12, 16). ERT with alglucosidase alfa was started on day 3 of life at a dose of 20 mg/kg followed by infusions once every 2 weeks as per package insert (13). The patient was discharged home from the NICU at 1 month of age and continued to receive ERT on an outpatient basis every 2 weeks.

The patient successfully completed four doses of rituximab at 22 days of life, nine doses of methotrexate at 32 days of life, and continued to receive monthly IVIG until 6 months of age. There were no infections around the time of ITI administration. The patient tolerated the ITI protocol safely with B-cell recovery, measured as CD19 count of 1,448 cells/μl (370–2,306 cells/μl), at 3 months of age. The patient started her immunization schedule at 9 months of age and antibody titers to Tetanus toxoid and Diphtheria checked at 18 months of age revealed an adequate response.

### Growth and Developmental Status

The patient has shown normal growth velocity and at 4 years of age is at the 90th percentile for both height and weight. Developmentally she was sitting by 6 months of age, measured at the 75-90% on the Alberta Infant Motor Scale at 8 months and walked independently by 11 months of age. Now at 50 months of age, she continues to grow and develop appropriately based on the Early Learning Accomplishment Profile (ELAP). Audiology evaluation revealed bilateral mixed hearing loss at 3 years of age and she has been fitted with hearing aids. She attends a regular prekindergarten class and does not require any special services.

### Current Clinical Status

**Figure 2:**
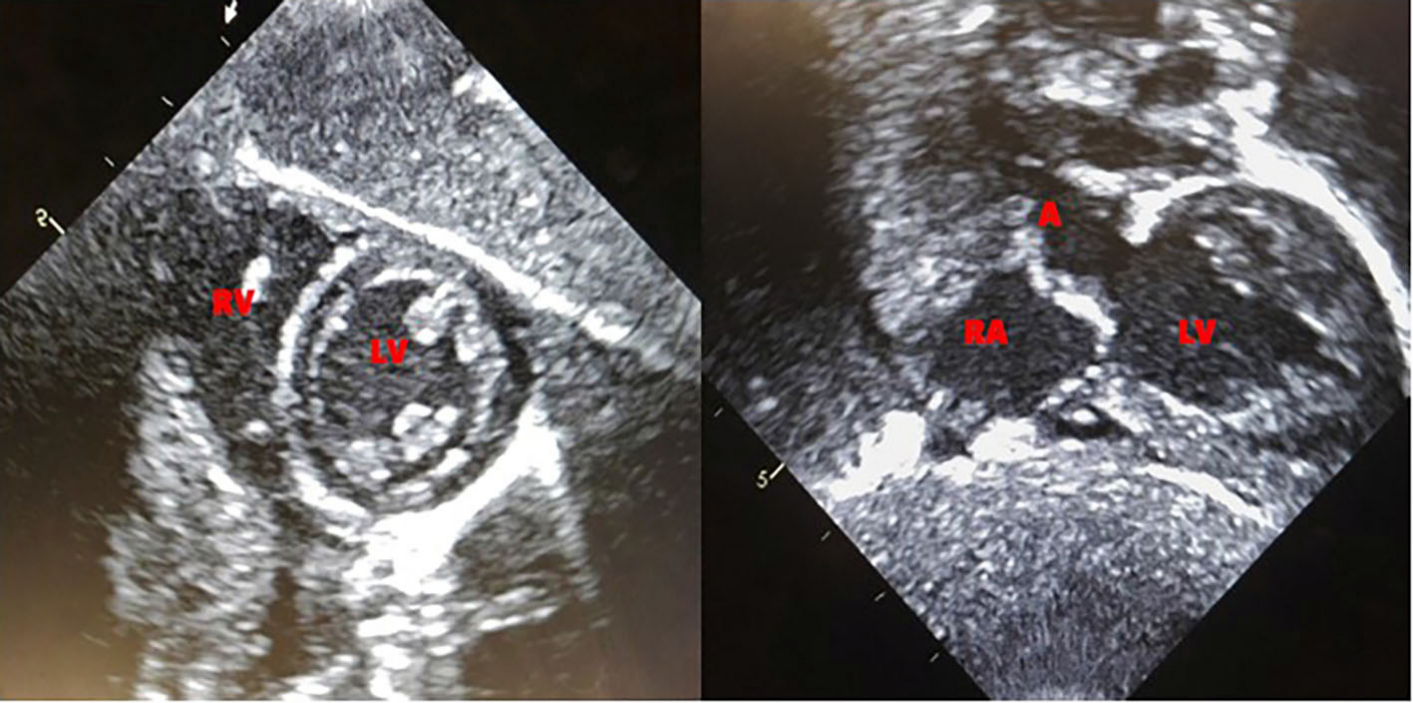
Echocardiogram at 11-weeks of life, showing resolution of ventricular hypertrophy.

**Figure 3:**
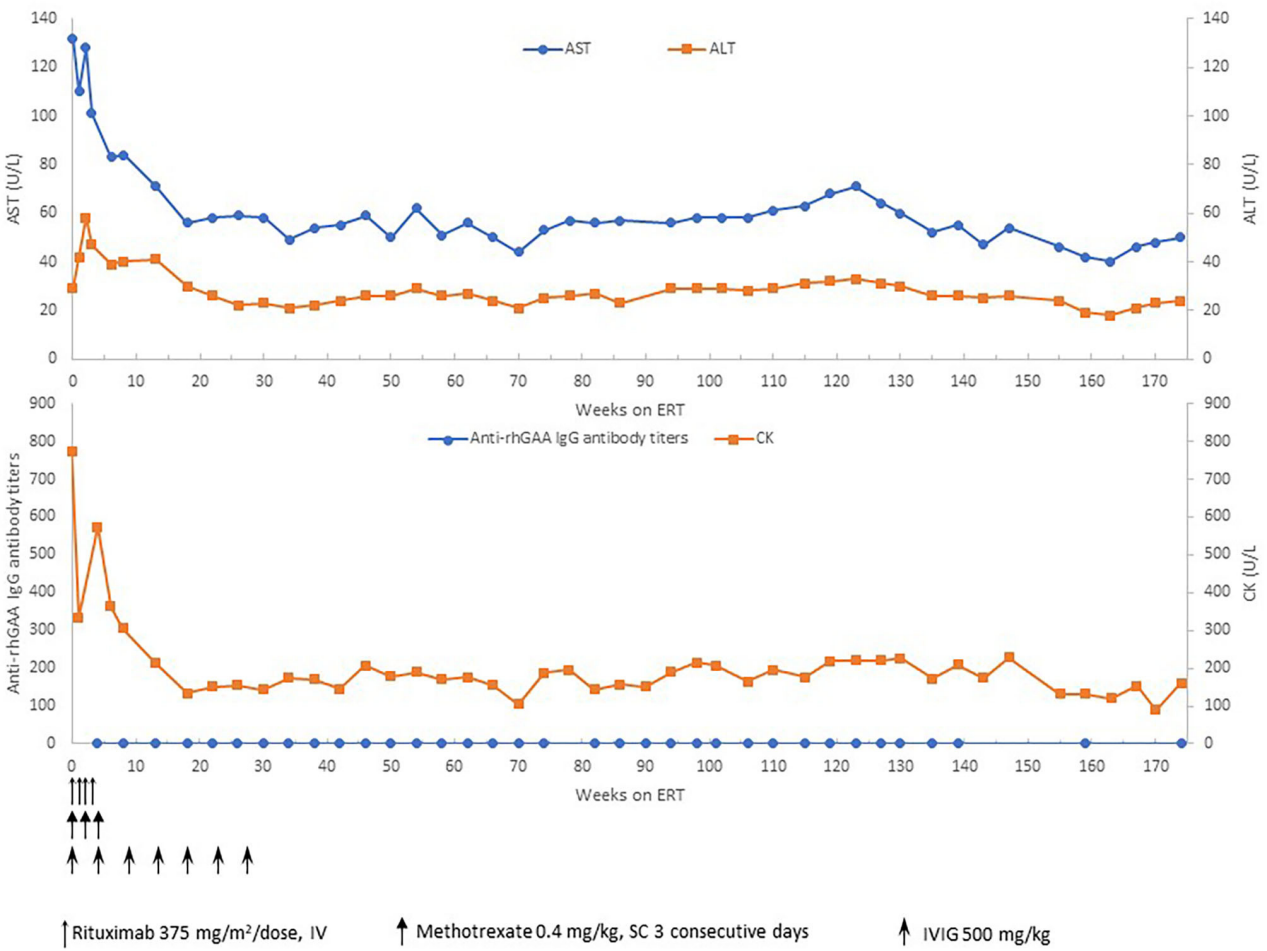
Anti-rhGAA IgG antibody titers, CK, AST, and ALT over time. ERT, enzyme replacement therapy; mg, milligram; kg, kilogram; CK, creatine kinase; AST, aspartate aminotransferase; ALT, alanine aminotransferase; U, units; L, liter.

Based on the published literature on apparent clinical benefits of increased ERT dose and concerns of clinical plateu in our patient, ERT dosing was increased to 40 mg/kg every 2 weeks at 32 months of age and frequency increased to 40 mg/kg every week at 47 months of age (17, 18). Complete recovery of cardiac function and left ventricular mass was seen by 11 weeks of age (Figure 2). No evidence of arrhythmia was seen based on a 24 h Holter monitor at 6 months of age (19). She continues to have undetectable anti-rhGAA antibody titers and normal urinary Hex4 at 50 months of age (20). Her AST, ALT, and total CKs have also been in the normal range (Figure 3). Patient is able to walk, run, skip, gallop, hop on one foot alternatingly, hop on both feet, and climb up and down the stairs independently.

The patient has not experienced any infusion associate reactions to the ERT to date. She continues to receive ERT at 40 mg/kg every week, has been off all ITI medications, continues to be seronegative, and is managed on an outpatient basis at a multispecialty center.

#### Current Respiratory Status

Patient has been diagnosed with reactive airway disease and has required albuterol nebulizations when symptomatic. She has undergone adenoidectomy due to a history of snoring. She never required invasive ventilation and remains on room air with no other respiratory support.

#### Current Gastrointestinal Status

The patient eats an age-appropriate diet by mouth with no assistance. She has regular bowel movements and attained bowel control by 40 months of age.

#### Current Cardiac Status

Patient continues to be followed by a pediatric cardiologist. Complete recovery of cardiac function and left ventricular mass was seen by 11 weeks of age (Figure 2). No evidence of arrhythmia was seen based on a 24-h Holter monitor at 6 months of age (19). Electrocardiogram and echocardiogram done every 3 months continue to show normal sinus rhythm and normal cardiac function, with the last one at 50 months of age.

## Discussion

Medical advances have come a long way in the past 20 years in our understanding of IPD. Once considered a fatal diagnosis, the advent of ERT has made it possible to significantly extend the life span of affected patients, which is largely attributable to improvements in cardiomyopathy and skeletal muscle function (21).

Amongst the prenatally diagnosed IPD cases Hamdan et al., in 2008 reported a prenatally diagnosed patient due to hypertrophic cardiomyopathy seen at 32 weeks fetal echocardiogram, diagnosis confirmed at birth by enzyme assay and mutation analysis which revealed a homozygous mutation for c.1327-2A>G. The infant was treated with ERT from 18 h of age and reportedly had a favorable outcome at 10 months. CRIM status was not determined and antibody titers were not reported (22).

Additionally, Abbot et al. (23) in 2011 reported a patient with prenatally diagnosed IPD due to family history with CRIM-negative (R854X/R854X) IPD, who received standard dosing of alglucosidase alfa (Myozyme®) enzyme replacement therapy (ERT) from day 10 of life until she passed away at the age of 3 years 9 months. In the immediate neonatal period, there was cardiomegaly on chest X-ray, cardiac hypertrophy by echocardiogram, and development of a wide complex tachycardia. The available data at the time indicated that CRIM-negative patients had limited survival even with ERT. However, given the opportunity for very early treatment, the treating provider and family elected to initiate treatment with ERT, without immune modulation. It was believed that the baby would not mount an immune response due to the immaturity of the developing immune system. By 9 months of age, an echocardiogram was normal. Early motor development was within normal limits but by 2 years of age, her developmental progress had slowed. She seroconverted by the 4th month of ERT, and anti-rhGAA antibody titers peaked at 25,600 in the 27th month and remained moderately elevated at 6,400 during the final 9 months of her life. Immunomodulatory therapy was considered but declined by family. She presented with cardiopulmonary arrest at 2 years 6 months and infection with Influenza A was confirmed. This led to a prolonged hospitalization with invasive respiratory support, and placement of tracheostomy and gastrostomy tube. Her developmental progress ceased, and she died suddenly at home from a presumed cardiac event at age 3 years 9 months.

Our patient is similar to the case described by Abbot et al. (23) with regards to the prenatal diagnosis of IPD as well as carrying the same homozygous *GAA* variant (R854X/R854X). This variant is one of the most frequently identified mutations in CRIM-negative alleles at up to 32.7% and commonly seen in the African American population. It is a nonsense mutation resulting in a premature stop codon (14). The key difference is that our patient was initiated on ITI in an ERT naïve setting at 2 days of life with rituximab, followed by ERT at 3 days of life. Our patient never developed an antibody response to rhGAA demonstrating tolerization to the ERT. Complete recovery of cardiac function and left ventricular mass was seen by 11 weeks of age. Respiratory assistance in the form of intermittent CPAP was required during the first 3 weeks of neonatal life, but invasive ventilation has never been required so far. She has met all developmental milestones appropriately and now at 50 months of age is attending a regular prekindergarten class. A prior study suggested that ERT initiation at a very early age (<2 months) may help to diminish anti-drug antibodies to ERT (9). However, as evident from case described by Abbot et al. (23), patient initiated on ERT even within few days of life, are still at risk of developing high and sustained antibodies to ERT, resulting in suboptiomal treatment response. In a retrospective study on CRIM-negative IPD treated with ERT monotherapy, Berrier et al. (5) described two CRIM-negative cases who were initiated on ERT within the 1st month of life and developed antibodies to ERT, leading to an eventual fatal outcome. Thus, the initiation of ERT at an early age does not prevent the development of antibodies to ERT.

To our knowledge, this is the youngest IPD patient initiated on immunomodulation with rituximab, methotrexate, and IVIG. The data on the safety of rituximab in the pediatric population is limited. Prior studies have demonstrated that rituximab can lead to skewing of B cell subpopulation, persistent hypogammaglobulinemia, and can affect the response to routine vaccination (24–29). These cases likely needed rituximab for a longer duration. The ITI protocol used in our patient requires only 4 doses of rituximab. In a large cohort of IPD patients, this short 5 week course of ITI was successful in inducing immune tolerance to ERT in 88% of CRIM-negative IPD patients (13). Additionally, ERT is given concurrently with ITI. This is different from other suggested protocols in literature which required delay in ERT initiation by 3 weeks for induction of immunomodulation; such delay in treatment initiation can negatively impact the long-term clinical outcome of IPD patients (30). Our patient tolerated ITI without any adverse events. She had full B cell reconstitution following completion of ITI and normal immunoglobulin levels at the most recent follow-up. She is up to date on age-appropriate vaccinations and demonstrated an adequate humoral response to routine vaccines.

The newborn screen program (NBS) has been a great public health achievement since its induction in the early 1960s but continues to evolve as medical advances make the diagnosis and treatment of certain conditions like IPD more feasible. Pompe Disease was formally included in the RUSP in March 2015 and has been added to the NBS in 21 states with additional states soon to follow (31). Although additional data may be necessary to understand the efficacy and efficiency of such universal screening practices, knowing that outcomes can be significantly improved by early induction of current IPD therapies, supports the addition of Pompe disease screening in the NBS of other states.

Our case exemplifies the integration of prenatal genetic diagnosis to the coordination of complex multidisciplinary care in the treatment of a rare, previously fatal genetic condition. Our patient has thus far tolerated the therapy well. Our experience with planning for and the management of this patient as well as the clinical outcome will provide crucial information especially in light of the addition of Pompe disease to the RUSP. As an increasing number of states now screen for Pompe disease in the newborn setting, there must be no delay in the timely initiation of appropriate treatment and the use of ITI as indicated.

## Ethics Statement

Written informed consent was obtained from the minor(s)' legal guardian/next of kin for the publication of any potentially identifiable images or data included in this article.

## Author Contributions

PG and BS wrote the manuscript. FK, DM, and JM performed fetal and postnatal cardiac measurements and collection of data. PT provided prenatal details. LT-K collected and organized lab results. AD made the graphs. Review and revision of the manuscript was done by PG, AD, and PK. All authors contributed to and approved the final manuscript.

## Conflict of Interest

PG has received consulting fees and honoraria from Sanofi Genzyme, Amicus Therapeutics, and Takeda Shire. BS received the 2019 Takeda Next Generation Medical Biochemical Subspeciality Fellowship Award. AD has received research support from Sanofi Genzyme and Lysosomal Disease Network (LDN). PK has received research/grant support from Sanofi Genzyme, Valerion Therapeutics, and Amicus Therapeutics. PK has received consulting fees and honoraria from Sanofi Genzyme, Amicus Therapeutics, Vertex Pharmaceuticals, and Asklepios Biopharmaceutical, Inc. (AskBio). PK is a member of the Pompe and Gaucher Disease Registry Advisory Board for Sanofi Genzyme, Amicus Therapeutics, and Baebies. PK has equity in Asklepios Biopharmaceutical, Inc. (AskBio), which is developing gene therapy for Pompe disease. The remaining authors declare that the research was conducted in the absence of any commercial or financial relationships that could be construed as a potential conflict of interest.
