# Supplementary material for: A Race Against Time—Changing the Natural History of CRIM Negative Infantile Pompe Disease
Source: Front Immunol. 2020 Sep 4;11:1929. doi: 10.3389/fimmu.2020.01929 (PMC7498628; doi:10.3389/fimmu.2020.01929)
Supplement: Supplementary file 1 [file Presentation_1.PPTX]

## Slide 1
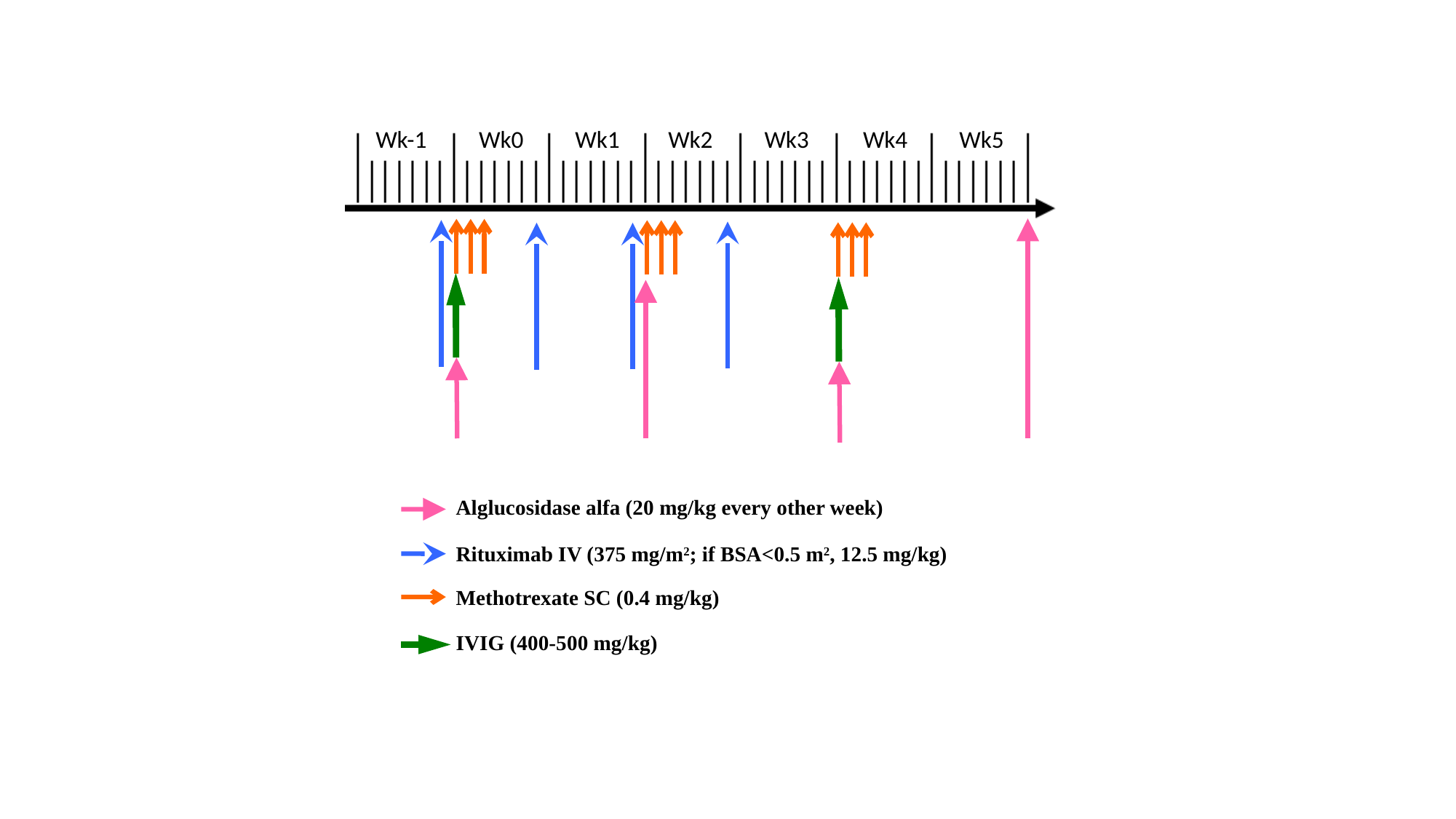

Wk-1
Wk0
Wk1
Wk2
Wk3
Wk4
Wk5
Alglucosidase alfa (20 mg/kg every other week)
Rituximab IV (375 mg/m2; if BSA<0.5 m2, 12.5 mg/kg)
Methotrexate SC (0.4 mg/kg)
IVIG (400-500 mg/kg)
